# Reliability, Validity and Temporal Stability of the Serbian Version of the Boston Carpal Tunnel Questionnaire

**DOI:** 10.3390/medicina58111531

**Published:** 2022-10-26

**Authors:** Darko Bulatovic, Dejan Nikolic, Marija Hrkovic, Tamara Filipovic, Dragana Cirovic, Natasa Radosavljevic, Milica Lazovic

**Affiliations:** 1 Institute for Rehabilitation, 11000 Belgrade, Serbia; 2 Faculty of Medicine, University of Belgrade, 11000 Belgrade, Serbia; 3 Department of Physical Medicine and Rehabilitation, University Children’s Hospital, 11000 Belgrade, Serbia; 4 Department of Biomedical Sciences, State University of Novi Pazar, 36300 Novi Pazar, Serbia

**Keywords:** carpal tunnel syndrome, Boston carpal tunnel questionnaire, validation, adults

## Abstract

*Background and Objectives*: The aim of this study was to validate the Serbian version of the Boston Carpal Tunnel Questionnaire (BCTQ) and to evaluate temporal stability for the purpose of its implementation in the evaluation of Serbian patients with carpal tunnel syndrome (CTS). *Materials and Methods*: For the validation of the Serbian version of the BCTQ (BCTQ_SR_), we tested 69 individuals with diagnosed CTS that were referred for a conservative treatment at the Institute for Rehabilitation. Neurophysiological tests were used for the electrophysiological grading (EG) of CTS severity in the study sample. The final version of the BCTQ_SR_ was given to the tested participants from the study on two occasions: test and retest, with a five-day period between the two measurements. *Results*: The mean value for the symptom severity subscale (SSS) of the BCTQ_SR_ was 3.01 ± 0.94; for the functional status subscale (FSS) of the BCTQ_SR_ it was 2.85 ± 1.00. Cronbach’s α for the SSS was 0.91 and 0.93 for the FSS. The intraclass correlation coefficients (ICCs) concerning the test–retest were significant (*p* < 0.001) and were 0.949 for the SSS and 0.959 for the FSS. Those with a higher EG grade had higher values of the SSS and FSS but without a statistical significance (*p* = 0.103 and *p* = 0.053, respectively). The intercorrelation of the BCTQ_SR_ subscales (SSS and FSS) on the test was significant (*p* < 0.001) with a correlation coefficient equal to 0.777. *Conclusion*: The Serbian version of the BCTQ (BCTQ_SR_) was successfully culturally adopted. The BCTQ_SR_ was a valid and reliable instrument for the measurement of symptom severity and functional status in adults with CTS. Therefore, it can be used in clinical practice for patients with CTS.

## 1. Introduction

Carpal tunnel syndrome (CTS) is considered to be the most common peripheral nerve entrapment syndrome [1,2]. It is caused by median nerve compression in the wrist region [2]. In the United States (US), the prevalence of the condition is 7.8% [3]; in European population studies, the prevalence ranges between 1 and 7% [4]. In Italy, for those who perform manual work, reports have stated an increase of over 170% in CTS between 2006 and 2010 [3]. Previously, it was stated that CTS is multifactorial with occupational risk factors such as repetitive hand movements, manual forceful exertion, hand–arm transmitted vibration and the bending or twisting of the wrist. Non-occupational risk factors include obesity, thyroid disease, pregnancy, diabetes mellitus, primary amyloidosis and rheumatoid arthritis, which can participate in the development of CTS [5]. Furthermore, it has been noticed that the body mass index (BMI) is an independent risk factor for CTS [6]. It is 3–4 times more likely for women to develop CTS than men and in 50% of cases with CTS both wrists are affected [7]. In the study of Farioli et al., it was stated that several epidemiological studies had been performed in order to evaluate the gender-specific causes of CTS, including hormonal factors, anthropometric parameters, pregnancy and non-occupational biomechanical exposure [8].

Even though CTS is primarily a clinical diagnosis [9], the confirmation is obtained by electrodiagnostic (EDX) studies [10]. Moreover, EDX studies are useful in a severity assessment of CTS and surgery planning [11]; however, the electrodiagnostic CTS severity might not be associated with the clinical severity [12]. The importance of a prompt and adequate diagnosis in patients with CTS is due to the fact that misdiagnosis and delays in establishing a diagnosis can lead to the persistence of symptoms and prolonged functional impairments [13].

Karabinov et al., stated that numerous questionnaires have been developed for the evaluation of upper limb disease, but the Boston Carpal Tunnel Questionnaire (BCTQ) is used most frequently as a disease-specific instrument for CTS [14]. The BCTQ is a patient-based outcome measure of symptom severity and functional status, specifically developed for CTS patients [15]. So far, the BCTQ has been validated in many languages, including Greek, Bulgarian, Dutch, Chinese, Portuguese, Turkish, Korean, Spanish, Finnish, Slovak and Arabic [12,14,16,17,18,19,20,21,22,23,24]. The BCTQ is a self-administered questionnaire and, as such, might eliminate bias and is sensitive to clinical changes even though it is subjective [19]. Furthermore, in the secondary analysis of Jerosch-Herold et al., it was pointed out that the symptom severity subscale (SSS) and functional status subscale (FSS) of the BCTQ should be evaluated as two separate subscales instead of being summed into a total score [25].

The aim of this study was to validate the Serbian version of the BCTQ and to evaluate its temporal stability for the purpose of the implementation of this questionnaire in the evaluation of Serbian patients with CTS.

## 2. Materials and Methods

### 2.1. Study Group

For the validation of the Serbian version of the BCTQ (BCTQ_SR_), we tested 69 individuals with diagnosed CTS that were referred for a conservative treatment at the Institute for Rehabilitation. The diagnosis of CTS was made by a board-certified Physical Medicine and Rehabilitation (PM&R) specialist with experience in CTS diagnostics and treatments. The inclusion criteria were native Serbian language-speaking patients with a first-time CTS diagnosis. The exclusion criteria were: age under 18 years; the presence of diabetes mellitus, rheumatoid arthritis, polyneuropathy, pregnancy, hypothyroidism and cervical radiculopathy; and cognitively challenged patients who were unable to fill in the questionnaire. Further variables analyzed were gender, age, occupation, dominant hand, lateralization of symptoms and electrophysiological grading on the right and left hand. Prior to inclusion in the study, the participants were informed and consent was obtained. The study was approved by the Institutional Review Board (No: 02/942-2, 13 September 2022).

### 2.2. Electrophysiological Grading

Neurophysiological tests were used for the electrophysiological grading (EG) of CTS severity in the study sample. Grade 0 referred to the absence of neurophysiological abnormalities; Grade 1 or very mild CTS were described as present abnormalities only in two sensitive tests, including a palm/wrist median/ulnar comparison, inching and a ring-finger “double peak”; Grade 2 or a mild degree of CTS were referred to as the presence of orthodromic sensory conduction velocity from the index finger to the wrist below 40 m/s along with a median motor terminal latency from the wrist to the abductor pollicis brevis muscle below 4.5 ms; Grade 3 or a moderately severe type of CTS were described if the motor terminal latency of the median nerve was above 4.5 ms and lower than 6.5 ms with a preserved sensory nerve action potential from the index finger; Grade 4 or severe CTS were noticed if the motor terminal latency of the median nerve was above 4.5 ms and below 6.5 ms as well as an absent sensory nerve action potential; Grade 5 or very severe CTS were referred to for those with a motor terminal latency of the median nerve above 6.5 ms; and Grade 6 or an extremely severe type of CTS were described if the surface motor potential from the abductor pollicis brevis muscle was below 0.2 mV, peak-to-peak [26].

### 2.3. Boston Carpal Tunnel Questionnaire

The BCTQ is a self-administered instrument composed of two subscales; one measures the severity of the symptoms and the other measures the functional status [12,17,24]. The symptom severity subscale (SSS) consists of 11 items assessing pain, paresthesia, numbness, weakness, nocturnal symptoms and overall functional status. The functional status subscale (FSS) consists of 8 items that assess the hand function during common daily activities. Every item scores between 1 and 5: SSS 1 is considered to be no symptoms and 5 is the worst symptoms; FSS 1 is considered to be no difficulty and 5 is an inability to perform activities at all. The overall SSS and FSS scores are calculated as the mean of the scores for the 11 and 8 individual items, respectively, where higher final scores point to a worse condition representation of the patient.

### 2.4. Adaptation Process

For the purpose of the translation and cultural adaptation of the BCTQ to the BCTQ_SR_ we followed the recommendations of the American Association of Orthopedic Surgeons (AAOS) [27]. At the initial stage or the forward translation, we engaged two bilingual translators of different profiles and backgrounds whose first language was Serbian to produce two translated versions (T1 and T2). One translator was aware of the concepts being examined in the translated questionnaire whereas the other was neither aware nor informed. At the second stage or the translation synthesis, a bilingual board-certified PM&R specialist synthesized the T1 and T2 translated versions, along with the two translators who had participated in the forward translation. At this stage, an active discussion took place regarding any potential discrepancies, finally reaching a consensus and producing a common version of the translation: T12. At the third stage or the stage of back translation, two bilingual translators with English as their mother tongue were engaged; they were neither aware nor informed of the explored concepts and produced two back translated versions (BT1 and BT2). At the fourth stage, an expert committee was formed to achieve cross-cultural equivalence. The expert committee was composed of two university professors of PM&R and two active specialists of PM&R with clinical practice of more than 5 years and with expertise in CTS as well as the bilingual translators included in forward and back translation processes to achieve a consensus and produce a pre-final version of the BCTQ_SR_. At the fifth stage, the pre-final version of the BCTQ_SR_ was distributed to 15 participants who had been diagnosed with CTS. All feedback was discussed and solved, producing the final version of the BCTQ_SR_ [27]. The final version of the BCTQ_SR_ was given to the test participants from the study on two occasions: a test and a retest, with a five-day period between the two measurements.

### 2.5. Statistical Analysis

The results were presented as numbers (N) and percentages (%) for the categorical variables and mean values (MV) with a standard deviation (SD) for the continuous variables. Cronbach’s α was used to assess the internal consistency. For the test–retest reliability, we used the intraclass correlation coefficient (ICC). Values of Cronbach’s α above 0.70 were considered to be acceptable [12]. Reliability, according to the values of the ICCs, was grouped as >0.90 = high, 0.75–0.90 = good, 0.50–0.75 = moderate and <0.50 = poor [28]. The test–retest reliability was further analyzed by Bland–Altman plots. The Pearson correlation coefficient was used to assess the intercorrelations of the SSS and FSS subscales as well as to correlate the subscales with an age. Differences between the subscales, according to the gender and EG grading, were obtained by an independent sample test. A receiver operating characteristic (ROC) curve was used to assess the ability of the subscales to discriminate between individuals with a low EG and those with a high EG. The performance was analyzed by the area under the curve (AUC). The statistical significance was set at *p* < 0.05.

## 3. Results

The characteristics of the patients are presented in Table 1. Female patients were predominantly represented (85.51%). An office job (46.38%) was the most frequent in the study sample regarding the occupation type. The right-handed were predominant (95.65%) and the localization of symptoms symmetrically on both sides was present in half of the individuals tested (50.72%). Considering the electrophysiological grading, the most frequent was Grade 2 (45.45%), followed by Grade 3 (43.93%) on the right side. The same applied for the left side, where Grade 2 was present in 37.68% and Grade 3 in 30.44% (Table 1). Three patients with bilateral CTS were excluded when the electrophysiological grading of the right hand was performed due to a surgical treatment for CTS on the right hand.

The mean value for the SSS of the BCTQ_SR_ was 3.01 ± 0.94; for the FSS of the BCTQ_SR_ it was 2.85 ± 1.00. Cronbach’s α for the SSS was 0.91; for the FSS, it was 0.93. These represented an acceptable internal consistency. All items for the SSS and FSS were above 0.70 (Table 2).

The ICCs concerning the test–retest were significant (*p* < 0.001); these were 0.949 for the SSS and 0.959 for the FSS.

**Figure 1:**
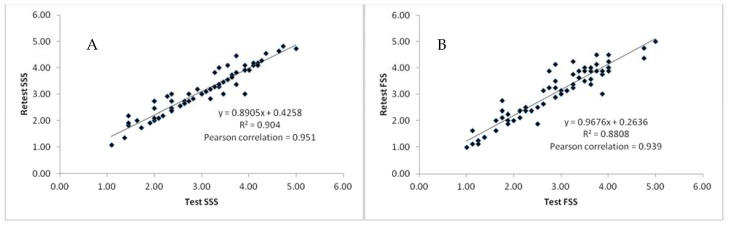
Scatterplot of the test and retest of the symptom severity subscale (SSS) and functional status subscale (FSS) of the Boston Carpal Tunnel Questionnaire Serbian Version (BCTQ_SR_). (**A**): Scatterplot and Pearson’s correlation of the test and retest of the SSS. (**B**): Scatterplot and Pearson’s correlation of the test and retest of the FSS.

The scatterplot graphs are presented in Figure 1. There was a high correlation between the test and the retest using Pearson’s correlation both for the SSS (0.951) and the FSS (0.939) (Figure 1).

**Figure 2:**
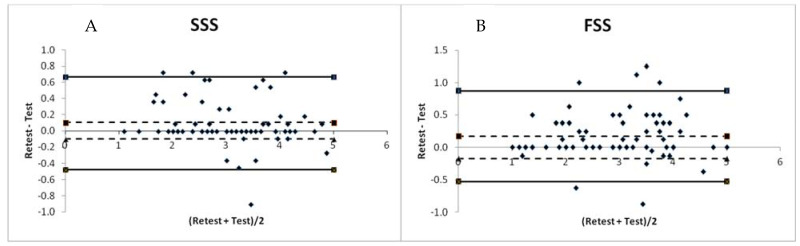
Bland–Altman plots of the test and retest of the symptom severity subscale (SSS) and functional status subscale (FSS) of the Boston Carpal Tunnel Questionnaire Serbian Version BCTQ_SR_. (**A**): Bland–Altman plot of the test and retest of the SSS. (**B**): Bland–Altman plot of the test and retest of the FSS. - - - - negative and positive average difference; - negative and positive 95% confidence interval.

The Bland–Altman plots are presented in Figure 2. The limits of agreement (LoA) for the SSS varied from −0.47 (with 95% CI from −0.36 to −0.58) to 0.67 (with 95% CI from 0.51 to 0.83). For the FSS, the total score varied from −0.53 (with 95% CI from −0.41 to −0.65) to 0.87 (with 95% CI from 0.67 to 1.07) for the time interval between the test and retest, suggesting an acceptable agreement between these two measurements. The average difference for the SSS was 0.10 (with 95% CI from 0.08 to 0.12) and 0.17 for the FSS (from 95% CI from 0.13 to 0.21).

The SSS and FSS scores were higher in females but without a statistical significance (*p* = 0.643 and *p* = 0.741, respectively) on the test. Those with a higher EG grade had higher values of the SSS and FSS but without a statistical significance (*p* = 0.103 and *p* = 0.053, respectively) on the test. Furthermore, there were non-significant correlations between gender and the SSS (*p* = 0.719) and FSS (*p* = 0.284) on the test (Table 3).

**Figure 3:**
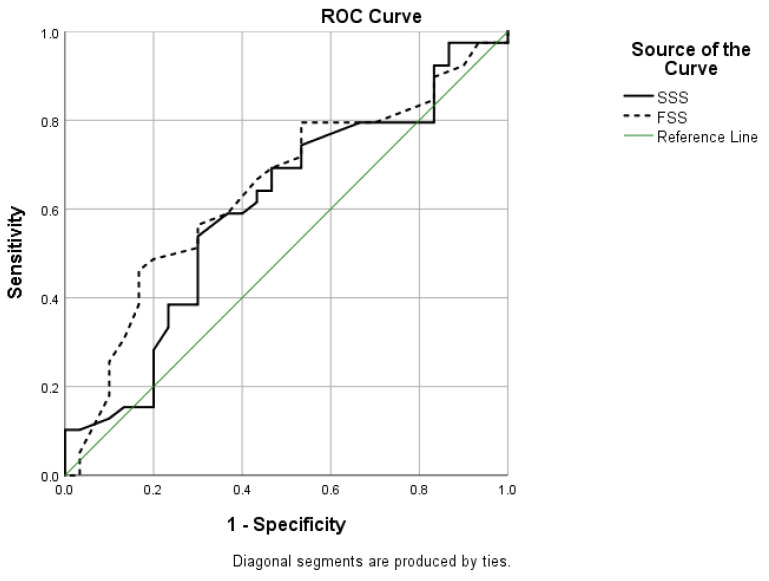
Receiver operating characteristic (ROC) curve for the prediction of a high electrophysiological grading (EG) for symptom severity subscale (SSS) and functional status subscale (FSS) of Boston Carpal Tunnel Questionnaire Serbian Version (BCTQ_SR_) on the test.

A ROC curve analysis demonstrated that for the SSS, the cut-off value was 3.32, with a sensitivity of 53.8%, a specificity of 70% and an AUC of 0.603 (*p* = 0.146). For the FSS, the cut-off value was 3.06, with a sensitivity of 56.4%, a specificity of 70% and an AUC of 0.644 (*p* = 0.042) (Figure 3).

The intercorrelation of the BCTQ_SR_ subscales (SSS and FSS) on the test was significant (*p* < 0.001), with a correlation coefficient equal to 0.777.

There was a significant correlation between the SSS items on the test, except for the correlations between SSS Item 6 and SSS Item 2 (r = 0.192; *p* = 0.114), SSS Item 6 and SSS Item 4 (r = 0.189; *p* = 0.121), SSS Item 6 and SSS Item 5 (r = 0.062; *p* = 0.610), SSS Item 8 and SSS Item 5 (r = 0.216; *p* = 0.074) and SSS Item 10 and SSS Item 5 (r = 0.184; *p* = 0.131). The highest correlation was between Item 5 and Item 4 (r = 0.858; *p* < 0.001) and the lowest correlation was between Item 6 and Item 5 (r = 0.062; *p* = 0.610) (Table 4).

There was a significant correlation between all FSS items on the test (*p* < 0.001), with the highest correlation between Item 7 and Item 6 (r = 0.833; *p* < 0.001) and the lowest between Item 7 and Item 1 (r = 0.443; *p* < 0.001) (Table 5).

## 4. Discussion

The translated version of the BCTQ_SR_ was successful because only minor cultural adaptions were needed. The BCTQ_SR_ demonstrated a satisfactory internal consistency and test–retest reliability, with an acceptable agreement between the test and retest for both the SSS and FSS subscales. Furthermore, there was a significant intercorrelation between the BCTQ_SR_ SSS and FSS subscales on the test session.

Regarding the cultural adaptation of the BCTQ_SR_ SSS and FSS items, we also considered the observations from the study of Mendoza-Pulido and Ortiz-Corredor, where weaknesses such as fatigue, sleepiness, unsteadiness or loss of muscle strength could be widely interpreted in patients [29].

The importance of a satisfactory internal consistency refers to the fact that a higher internal consistency is associated with a greater precision or a lower error variance [19]. The results of our study regarding the internal consistencies for the BCTQ_SR_ SSS (Cronbach’s α = 0.91) and for the BCTQ_SR_ FSS (Cronbach’s α = 0.93) were in line with previous reports. For example, in the Spanish BCTQ validation for the SSS, the Cronbach’s α was 0.909 and for the FSS, the Cronbach’s α was 0.872 [21]. In the Greek BCTQ validation, the Cronbach’s α was 0.89 for the SSS and the Cronbach’s α was 0.93 for the FSS [12]. In the Dutch validation, it was somewhat lower for the SSS (Cronbach’s α = 0.847) and for the FSS (Cronbach’s α = 0.825) [16].

In our study, we had no loss of participants between the test and retest of the BCTQ_SR_. Furthermore, no incomplete questionnaires were returned both for the SSS and the FSS on the test and retest. In the study of Leite et al., it was stated that the BCTQ was shown to have good levels of acceptability, with response rates of 90% and above [15].

Even though we found no significant differences in the SSS and FSS scores of the BCTQ_SR_ between genders, females with CTS had higher scores on both BCTQ_SR_ subscales. Our findings differed somewhat when compared with previously reported results, where females with CTS had significantly higher values of both the SSS and FSS [12]. Despite the possibility that females might have a higher sensitivity in the reporting of CTS symptoms and that men might possibly have a higher tolerance for the symptoms [30], the possible explanation for our findings might be in the different cultural and social environment. Furthermore, gender was shown not to significantly correlate with the SSS and FSS of the BCTQ_SR_.

The BCTQ_SR_ scores in our study were higher for patients with CTS who were graded three and above on the EG versus those who were graded from zero to two, but this was without a statistical significance. In the Greek validation, the authors demonstrated significantly increased values for those with Grade 3 and above on the EG when compared with those who had Grades 1 and 2 [12]. The possible explanation for the absence of a significant difference between the tested groups of patients in our study might be due to different perceptions of symptom severity as well as different perceptions of functional changes in the tested patients with CTS.

In the Rash analysis of Multanen et al., regarding the structural validity of the BCTQ, it was noticed that the BCTQ SSS demonstrated multidimensionality whereas the FSS showed a unidimensional structure [22]. Furthermore, these authors pointed to the fact that the question in the BCTQ SSS “How long on average does an episode of pain last during the daytime” demonstrated a non-uniform differential item functioning that favored age whereas Item 7 was shown to favor gender [22].

When considering the correlation between the different items in the BCTQ_SR_ SSS on the test session and the possible explanation for the absence of statistical significances between certain items of the SSS, we referred to the fact that, according to De Kleermaeker et al., the FSS could be considered to be a unidimensional scale whereas the SSS subscale measures three different factors (daytime symptoms, night-time symptoms and operational capacity) [16]. Assuming this, the absence of significant correlations between Item 6 and Item 4, between Item 6 and Item 5 and between Item 8 and Item 5 as well as between Item 10 and Item 5 for the SSS could be explained by the possibility that they belonged to different factors such as “daytime symptoms” for Items 4 and 5 and “night-time symptoms and numbness/tingling” for Item 6, Item 8 and Item 10, as stated in the study of Atroshi et al. [31].

## 5. Conclusions

The Serbian version of the BCTQ (BCTQ_SR_) was successfully culturally adopted. The BCTQ_SR_ was a valid and reliable instrument for the measurement of symptom severity and functional status in adults with CTS. Thus, it can be used in clinical practice for patients with CTS.

## Figures and Tables

**Table 1:** Patient characteristics.

Gender (*n* = 69)	
Male, *n* (%)	10 (14.49)
Female, *n* (%)	59 (85.51)
Age (MV ± SD)	55.67 ± 10.77
Occupation (*n* = 69)	
Physical job, *n* (%)	20 (28.98)
Office job, *n* (%)	32 (46.38)
Unemployed, *n* (%)	6 (8.70)
Retired, *n* (%)	11 (15.94)
Dominant Hand (*n* = 69)	
Right, *n* (%)	66 (95.65)
Left, *n* (%)	1 (1.45)
Ambidextrous	2 (2.90)
Lateralization of Symptoms (*n* = 69)	
Both sides, symmetrically, *n* (%)	35 (50.72)
Both sides, more right, *n* (%)	17 (24.64)
Both sides, more left, *n* (%)	6 (8.70)
Only right side, *n* (%)	10 (14.49)
Only left side, *n* (%)	1 (1.45)
Electrophysiological Grading: Right Hand (*n* = 66)	
Grade 0, *n* (%)	1 (1.52)
Grade 1, *n* (%)	1 (1.52)
Grade 2, *n* (%)	30 (45.45)
Grade 3, *n* (%)	29 (43.93)
Grade 4, *n* (%)	2 (3.03)
Grade 5, *n* (%)	1 (1.52)
Grade 6, *n* (%)	2 (3.03)
Electrophysiological Grading: Left Hand (*n* = 69)	
Grade 0, *n* (%)	10 (14.49)
Grade 1, *n* (%)	9 (13.04)
Grade 2, *n* (%)	26 (37.68)
Grade 3, *n* (%)	21 (30.44)
Grade 4, *n* (%)	2 (2.90)
Grade 5, *n* (%)	1 (1.45)
Grade 6, *n*(%)	0

MV—Mean value; SD—Standard deviation.

**Table 2:** Mean values of the BCTQ_SR_ and Cronbach’s α values.

BCTQ_SR_ Items	MV ± SD	Cronbach’s α if Item Deleted	Total Cronbach’s α
SSS			
1	3.01 ± 1.37	0.89	-
2	2.94 ± 1.43	0.90	-
3	2.74 ± 1.18	0.90	-
4	3.07 ± 1.36	0.90	-
5	2.83 ± 1.39	0.91	-
6	3.28 ± 1.23	0.91	-
7	2.90 ± 1.20	0.90	-
8	3.13 ± 1.25	0.91	-
9	3.45 ± 1.27	0.90	-
10	3.17 ± 1.33	0.90	-
11	2.59 ± 1.28	0.90	-
Total	3.01 ± 0.94	-	0.91
FSS			
1	2.43 ± 1.24	0.93	-
2	2.61 ± 1.20	0.91	-
3	2.81 ± 1.29	0.92	-
4	2.86 ± 1.25	0.92	-
5	3.22 ± 1.29	0.92	-
6	3.19 ± 1.09	0.91	-
7	3.28 ± 1.22	0.92	-
8	2.39 ± 1.20	0.92	-
Total	2.85 ± 1.00	-	0.93

BCTQ_SR_—Boston Carpal Tunnel Questionnaire Serbian Version; MV—Mean value; SD—Standard deviation; SSS—Symptom severity subscale; FSS—Functional status subscale.

**Table 3:** BCTQ_SR_ association of subscales (SSS and FSS) with gender, age and EG grading on the test.

Tested Variables	SSS		FSS	
		*p*-Value		*p*-Value
Gender				
Male (MV ± SD)	2.88 ± 0.99	0.643 *	2.75 ± 1.18	0.741 *
Female (MV ± SD)	3.03 ± 0.94		2.86 ± 0.98	
Age				
r **	−0.044	0.719 **	0.131	0.284 **
EG				
1–2 (MV ± SD)	2.80 ± 0.91	0.103 *	2.58 ± 0.97	0.053 *
≥3 (MV ± SD)	3.17 ± 0.94		3.05 ± 0.99	

MV—Mean value; SD—Standard deviation; SSS—Symptom severity subscale; FSS—Functional status subscale; EG—Electrophysiological grading; * Independent sample test; ** Pearson correlation coefficient.

**Table 4:** Correlations between SSS items on the test.

Correlations												
BCTQ_SR_ SSS		Item 1	Item 2	Item 3	Item 4	Item 5	Item 6	Item 7	Item 8	Item 9	Item 10	Item 11
Item 1	r	1										
	*p*											
Item 2	r	0.811	1									
	*p*	<0.001										
Item 3	r	0.639	0.606	1								
	*p*	<0.001	<0.001									
Item 4	r	0.630	0.483	0.731	1							
	*p*	<0.001	<0.001	<0.001								
Item 5	r	0.534	0.385	0.605	0.858	1						
	*p*	<0.001	0.001	<0.001	<0.001							
Item 6	r	0.346	0.192	0.392	0.189	0.062	1					
	*p*	0.004	0.114	0.001	0.121	0.610						
Item 7	r	0.645	0.543	0.684	0.533	0.428	0.484	1				
	*p*	<0.001	<0.001	<0.001	<0.001	<0.001	<0.001					
Item 8	r	0.465	0.333	0.382	0.314	0.216	0.549	0.440	1			
	*p*	<0.001	0.005	0.001	0.009	0.074	<0.001	<0.001				
Item 9	r	0.659	0.598	0.374	0.304	0.245	0.493	0.455	0.558	1		
	*p*	<0.001	<0.001	0.002	0.011	0.042	<0.001	<0.001	<0.001			
Item 10	r	0.598	0.747	0.432	0.261	0.184	0.347	0.389	0.439	0.731	1	
	*p*	<0.001	<0.001	<0.001	0.030	0.131	0.003	0.001	<0.001	<0.001		
Item 11	r	0.493	0.461	0.611	0.440	0.456	0.408	0.730	0.450	0.397	0.398	1
	*p*	<0.001	<0.001	<0.001	<0.001	<0.001	0.001	<0.001	<0.001	0.001	0.001	

BCTQ_SR_—Boston Carpal Tunnel Questionnaire Serbian Version; SSS—Symptom severity subscale. r: Pearson’s correlation.

**Table 5:** Correlations between FSS items on the test.

Correlations									
BCTQ_SR_ FSS		Item 1	Item 2	Item 3	Item 4	Item 5	Item 6	Item 7	Item 8
Item 1	r	1							
	*p*								
Item 2	r	0.666	1						
	*p*	<0.001							
Item 3	r	0.512	0.731	1					
	*p*	<0.001	<0.001						
Item 4	r	0.457	0.596	0.777	1				
	*p*	<0.001	<0.001	<0.001					
Item 5	r	0.462	0.556	0.591	0.592	1			
	*p*	<0.001	<0.001	<0.001	<0.001				
Item 6	r	0.471	0.708	0.687	0.646	0.702	1		
	*p*	<0.001	<0.001	<0.001	<0.001	<0.001			
Item 7	r	0.443	0.654	0.631	0.622	0.547	0.833	1	
	*p*	<0.001	<0.001	<0.001	<0.001	<0.001	<0.001		
Item 8	r	0.681	0.798	0.609	0.507	0.597	0.662	0.675	1
	*p*	<0.001	<0.001	<0.001	<0.001	<0.001	<0.001	<0.001	

BCTQ_SR_—Boston Carpal Tunnel Questionnaire Serbian Version; FSS—Functional status subscale. r: Pearson’s correlation.

## Data Availability

Original data are available on request from the first author (D.B.)
